# Clinical and prognostic implications of rim restriction following glioma surgery

**DOI:** 10.1038/s41598-022-16717-y

**Published:** 2022-07-27

**Authors:** Assaf Berger, Garry Gali Tzarfati, Marga Serafimova, Pablo Valdes, Aaron Meller, Akiva Korn, Naomi Kahana Levy, Daniel Aviram, Zvi Ram, Rachel Grossman

**Affiliations:** 1 Department of Neurosurgery, Tel Aviv Medical Center, 6 Weizmann St., Tel Aviv, 6423906 Israel; 2 Division of Anesthesiology, Tel-Aviv University, Tel-Aviv, Israel; 3 Sackler Faculty of Medicine, Tel-Aviv University, Tel-Aviv, Israel; 4 Center for Advanced Radiosurgery, NYU Langone Medical Center, New York University, 530 First Avenue, New York, NY 10016 USA

**Keywords:** CNS cancer, Stroke

## Abstract

Rim restriction surrounding the resection cavity of glioma is often seen on immediate post-op diffusion-weighted imaging (DWI). The etiology and clinical impact of rim restriction are unknown. We evaluated the incidence, risk factors and clinical consequences of this finding. We evaluated patients that underwent surgery for low-grade glioma (LGG) and glioblastoma (GBM) without stroke on post-operative imaging. Analyses encompassed pre- and postoperative clinical, radiological, intraoperative monitoring, survival, functional and neurocognitive outcomes. Between 2013 and 2017, 63 LGG and 209 GBM patients (272 in total) underwent surgical resection and were included in our cohort. Post-op rim restriction was demonstrated in 68 patients, 32% (n = 20) of LGG and 23% (n = 48) of GBM patients. Risk factors for restriction included temporal tumors in GBM (p = 0.025) and insular tumors in LGG (p = 0.09), including longer surgery duration in LGG (p = 0.008). After a 1-year follow-up, LGG patients operated on their dominant with post-op restriction had a higher rate of speech deficits (46 vs 9%, p = 0.004). Rim restriction on postoperative imaging is associated with longer duration of glioma surgery and potentially linked to brain retraction. It apparently has no direct clinical consequences, but is linked to higher rates of speech deficits in LGG dominant-side surgeries.

## Introduction

Maximal safe resection is the desired goal in surgery of both high- and low-grade gliomas, and it has also been linked to better overall survival^1–3^. However, postoperative deficits are still a major concern due to their association with impaired quality of life and even decreased survival^4,5^.

Diffusion-weighted imaging (DWI) restrictive changes are often seen transiently at the rim surrounding the resection cavity after surgery for gliomas^6^. Post-operative diffusion restriction is thought to result from cellular injury and swelling which decreases the movement of protons^7,8^. As opposed to intra-operative strokes which are occasionally demonstrated by post-operative DWI and have been discussed in depth in the past, the etiology and clinical impact of peri-resection rim restriction remain unclear^9–12^. Cases with this imaging finding have been either excluded from studies dealing with perioperative ischemic complications or incorporated into groups of combined ischemic lesions. Furthermore, these studies have often been composed of a variety of types of tumors^10,12–15^.

The purpose of this study was to characterize the various aspects of rim restrictions as depicted on imaging studies following resection of low grade glioma (LGG) and glioblastoma (GBM), including their incidence, risk factors, short- and long-term clinical, functional, and cognitive implications, as well as their associations with intraoperative events.

## Results

### Clinical and demographic data

Between 2013 and 2017, 468 patients underwent either surgical resection or biopsy at our center, of these 120 for LGG and 348 for GBM. Fourty-four cases of biopsy procedures alone (5 of LGG and 39 of GBM), as well as 23 patients who had no complete pre-and postoperative MRI data (4 of LGG and 19 of GBM), and 80 patients who had no detailed admission, surgery, and discharge or follow-up reports (29 of LGG and 51 of GBM) were excluded from the study. Nineteen LGG and 30 GBM patients who sustained an intraoperative stroke were also excluded. Our final cohort consisted of 272 patients (63 LGG and 209 GBM) who underwent resection and had full datasets that included a long-term clinical evaluation and no evidence of intra-operative stroke.

The combined population mean age at surgery was 53 ± 16 years, with LGG patients being significantly younger than those with GBM (37 ± 12 and 58 ± 14, respectively, p = 0.001). Of the combined study population, 102 (38%) were females with similar rates within each tumor study group. There were 100 (37%) awake operations and 92 (34%) recurrent operations. There was a significantly higher rate of temporal tumors among GBM patients (33% vs 11%, p = 0.001), while insular tumors were more common among LGG patients (21% Vs 9%, p = 0.013). Median preoperative KPS was significantly higher among LGG patients than in GBM (90, 70–100 vs 80, 30–100, p = 0.001). While pre-operative tumor volume was similar (34 ± 32 ml in LGG and 33 ± 25 ml in GBM, p = 0.750), EOR was significantly higher in the GBM group (96 ± 8%) vs 87 ± 11% in the LGG group (p = 0.001).

### Incidence and risk factors

Rim restriction was demonstrated on the postoperative DWI studies of 68 patients (25%), including 20 LGG and 48 GBM patients.

We did not find significant differences in the rate of post-op rim restriction between LGG and GBM patients (32% vs 23%, p = 0.184).

We found no significant association between postoperative rim restriction and the evidence of blood in the surgical cavity that was demonstrated on the postoperative CT of 30% of the restriction cases and 28% of the non-restricted ones in LGG (*p* = 0.864), as well as 51% of GBM patients with restriction vs 49% in those without restriction (p = 0.177).

A univariate analysis that compared patients with and without rim restriction with respect to demographic, clinical, and pathological parameters showed no significant differences between those with and without rim-restriction in the combined GBM + LGG cohort (Table 1). Among GBM patients, rim restriction was more common in tumors located in the temporal lobes (32% Vs 18% in non-temporal areas, p = 0.025) and non-significantly more common in LGG of insular location (54% vs. 24%, p = 0.09). The calculated risk (odds ratio, OR) for post-op rim restriction among temporal GBM cases was 1.74 (95% CI 1.09–4.10, p = 0.027).

**Table 1:** Clinical and demographic data of patients with and without post-operative rim-restriction on diffusion-weighted imaging, including the entire study population (n = 272), low-grade glioma subgroup (n = 63) and high-grade glioma subgroup (n = 209).

	Entire study population (n = 272)			Low-grade glioma (n = 63)			Glioblastoma (n = 209)		
	No restriction, n = 204 (74%)	Restriction, n = 68 (26%)	Sig	No restriction, n = 43 (68%)	Restriction, n = 20 (32%)	Sig	No restriction, N = 161 (77%)	Restriction, N = 48 (23%)	Sig
Age	53 ± 17	53 ± 15	0.734	36 ± 11	41 ± 13	0.152	58 ± 15	58 ± 13	0.824
Sex (female, %)	77 (38%)	25 (37%)	0.885	27 (63%)	13 (65%)	1.0	61 (38%)	18 (38%)	0.961
BMI (kg/m^2^)	26.3 ± 4.0	26.1 ± 4.3	0.657	24.9 ± 4.2	25.1 ± 4.6	0.930	26.7 ± 3.9	26.5 ± 4.0	0.819
Pre-op KPS ≥ 70, %	179 (88%)	59 (87%)	0.758	60 (98%)	21 (100%)	0.744	136 (85%)	39 (81%)	0.533
Ever smoking, %	26 (13%)	11 (16%)	0.475	7 (16%)	4 (20%)	0.732	19 (12%)	7 (15%)	0.608
Diabetes mellitus	25 (12%)	5 (7%)	0.264	2 (4.7%)	0	0.999	23 (14%)	5 (10%)	0.490
IHD	14 (7%)	6 (9%)	0.592	1 (2.3%)	0	0.999	13 (8%)	6 (13%)	0.349
Hypertension	61 (30%)	14 (21%)	0.137	2 (5%)	2 (10%)	0.586	57 (35%)	14 (29%)	0.423
Pre-op serum Hb, g/dL	13.6 ± 1.7	13.5 ± 1.7	0.443	13.8 ± 1.4	13.6 ± 1.3	0.617	13.6 ± 1.8	13.4 ± 1.7	0.470
Recurrent, %	72 (35%)	20 (29%)	0.375	11 (26%)	5 (25%)	1.0	64 (40%)	12 (25%)	0.06
Previous radiation, %	51 (25%)	11 (16%)	0.133	4 (9%)	1 (5%)	0.488	49 (30%)	8 (17%)	0.06
Awake	73 (36%)	27 (40%)	0.561	19 (44%)	11 (55%)	0.589	53 (33%)	15 (31%)	0.828
Tumor enhancement	177 (87%)	57 (84%)	0.545	20 (47%)	7 (37%)	0.583	160 (99%)	4 (98%)	0.361
Tumor volume pre-op, cc	33.3 ± 26.9	33.7 ± 28.6	0.915	35 ± 36	33 ± 25	0.848	32 ± 24	36 ± 30	0.448
Tumor volume post-op, cc	1.71 ± 3.016	2.67 ± 5.712	0.186	3.36 ± 4.429	3.84 ± 3.758	0.665	1.26 ± 2.34	2.19 ± 6.323	0.322
EOR, %	94.3 ± 9.2	94.2 ± 9.2	0.936	89 ± 11	87 ± 11	0.483	95.9 ± 7.7	96.5 ± 7.4	0.672
P53 + , %	87 (55%)	34 (60%)	0.550	20 (47%)	12 (60%)	0.612	68 (59%)	21 (57%)	0.799
IDH 1 + , %	44 (27%)	22 (35%)	0.240	28 (65%)	16 (80%)	0.377	13 (11%)	9 (21%)	0.096
Frontal	93 (46%)	31 (46%)	0.999	18 (42%)	7 (35%)	0.783	77 (48%)	22 (46%)	0.808
Insular	27 (13%)	5 (7%)	0.192	6 (14%)	7 (35%)	0.092	14 (9%)	5 (10%)	0.716
Temporal	50 (25%)	25 (37%)	0.050	4 (9%)	3 (15%)	0.669	46 (29%)	22 (46%)	0.025
Parietal	56 (28%)	18 (27%)	0.875	10 (23%)	1 (5%)	0.151	51 (32%)	12 (25%)	0.376
Occipital	16 (8%)	6 (9%)	0.797	2 (5%)	0	0.999	15 (9%)	5 (10%)	0.820

*BMI* body mass index, *KPS* Karnofsky performance status, *MRS *Modified Rankin Scale, *CVA/TIA* cerebrovascular accident/transient ischemic attack, *IHD* ischemic heart disease, *DVT* deep vein thrombosis, *Hb* hemoglobin, *EOR* extent of resection, *IDH *isocitrate dehydrogenase 1.

#### IOM

Full IOM of MEPs, anesthesiology and awake monitoring reports were available in 152, 267 and 98 of the combined study population (n = 272), respectively. Neither MEPs changes nor awake intraoperative monitoring parameters showed any significant associations with rim restriction. Duration of anesthesia in the combined GBM and LGG cohort was significantly longer among patients with rim restriction, when compared to those without it (306 ± 87 min and 276 ± 81 min, respectively, *P* = 0.009). The difference was even more pronounced in the LGG group analysis (375 ± 17 min vs 316 ± 13 min; p = 0.008), Table 2.

**Table 2:** Correlations between post-op rim restriction on diffusion-weighted imaging and intraoperative parameters in the entire study population, high-grade glioma subgroup and low-grade glioma subgroup: intraoperative monitoring, awake surgery monitoring and anesthesiology parameters (univariate analysis).

IOM	Entire study population			Low-grade glioma			Glioblastoma		
	IOM (n = 152)			IOM (n = 34)			IOM (n = 118)		
	No restriction (n = 116)	Restriction (n = 36)	Sig	No Restriction (n = 20)	Restriction (n = 14)	Sig	No restriction (n = 90)	Restriction (n = 28)	Sig
MEPs decline (%)	13 (11%)	5 (14%)	0.664	6 (30%)	3 (21%)	0.704	7 (7.8%)	2 (7.1%)	0.912

Awake monitoring	Awake monitoring (n = 98)			Awake monitoring (n = 30)			Awake monitoring (n = 68)		
	No restriction (n = 72)	Restriction (n = 26)	Sig	No Restriction (n = 19)	Restriction (n = 11)	Sig	No restriction (n = 53)	Restriction (n = 15)	Sig
In op language decline	15 (21%)	2 (8%)	0.129	6 (32%)	2 (18%)	0.424	9 (17%)	0	0.087

Anesthesiology parameters	Anesthesiology parameters (n = 267)			Anesthesiology parameters (n = 63			Anesthesiology parameters (n = 204)		
	No restriction (n = 201)	Restriction (n = 66)	Sig	No restriction (n = 43)	Restriction (n = 20)		No restriction (n = 158)	Restriction (n = 46)	
Anesthesia time ± SD, min	276 ± 81	306 ± 87	0.009	316 ± 13	375 ± 17	0.008	265 ± 77	276 ± 73	0.362
MAP < 65 ± SD, min	35 ± 43	39 ± 50	0.511	29 ± 5	47 ± 14	0.241	37 ± 45	36 ± 43	0.919
Baseline SBP ± SD, mmHg	131 ± 22	132 ± 20	0.810	123 ± 16	128 ± 23	0.407	134 ± 23	134 ± 19	0.929
Minimal SBP ± SD, mmHg	83 ± 16	86 ± 17	0.281	86 ± 16	85 ± 15	0.702	82 ± 16	86 ± 19	0.172

*IOM *intra-operative monitoring, *MEP* motor evoked potentials, *ASA* American Society of Anesthesiology score, *TIVA* total intra-venous anesthesia, *SBP* systolic blood pressure, *MAP* mean arterial pressure, *SEM* standard error of mean.

### Clinical, functional, and cognitive outcomes

#### Overall and progression-free survival

The mean overall survival time in the LGG group was 56 months (95% confidence interval 52–60), and there was no perioperative mortality. There was no difference in survival between those with and without post-op restriction (*p* = 0.117). Mean progression free survival (PFS) in the entire LGG group was 44 months (95% CI 37–50) with no significant differences between the subgroups (p = 0.449).

Mean overall survival in the entire GBM group was 22 months (95% confidence interval 19–25). Interestingly, patients with immediate post-op restriction had a non-significant trend towards increased survival (26 months, 95% CI 19–32) as compared to those without (20 months, CI 95% 17–24, p = 0.076). Mean PFS in the GBM group was 13 months (95% CI 10–15) with a non-significant trend towards longer period among patients with immediate post-op restriction (15, 95% CI 19–21), as compared to those without rim restriction (11 months, 95% CI 8–14, p = 0.065).

#### Motor deficits

New or worsening immediate post-op motor deficits among LGG occurred in 4/20 (20%) of those with post-op restriction, similar to the rate in the non-restriction group (10/43 ,23%, p = 0.772). The rates were 9/48 (19%) in patients with post-op rim restriction and 19/161 (12%) of those without restriction (p = 0.215) in the GBM sub-population. In either group, no significant changes were noted in the rate of motor deficits over time, Tables 3 and 4.

**Table 3:** Clinical outcomes in low-grade glioma (LGG) patients with (n = 20) and without post-operative rim restriction on diffusion weighted imaging up to 1 year after surgery (n = 43).

	KPS		MRS		Motor deficit (%)		Seizures (%)		Speech deficits (%)*	
	No restriction	Restriction	No restriction	Restriction	No restriction	Restriction	No restriction	Restriction	No restriction	Restriction
Pre-op	90 (70–100)	90 (80–100)	1 (0–3)	1 (0–2)	21%	10%	56%	65%	27%	36%
Immediate post-op					28%	25%	63%	65%	18%	63%
3 months post-op	100 (60–100)	90 (60–100)	1 (0–3)	1 (0–3)	19%	20%	65%	65%	14%	73%
6-months post-op	90 (70–100)	90 (80–100)	1 (0–2)	1 (0–2)	16%	10%	67%	65%	9%	46%
12 months post-op	90 (60–100)	90 (80–100)	1 (0–3)	1 (0–2)	19%	10%	58%	60%	9%	46%
Sig.*	0.193 (time 0.840)		0.160 (time 0.726)		0.567 (Time 0.266)		0.739 (time 0.927)		0.004 (time 0.23)	

Multivariate analysis generalized linear models, adjusting for insular lesions and duration of anesthesia.

*KPS* Karnofsky Performance Status, *MRS* Modified Rankin Scale.

*Speech deficits analysis was performed in a subgroup of dominant side surgeries (n = 33), comparing restriction (n = 11) vs non-restriction groups (n = 22).

**Table 4:** Clinical outcomes in glioblastoma patients with (n = 48) and without post-operative rim-restriction on diffusion-weighted imaging (n = 161) up to 6 months after surgery.

	KPS		MRS		Motor deficit (%)		Seizures (%)		Speech deficits (%)*	
	No restriction	Restriction	No restriction	Restriction	No restriction	Restriction	No restriction	Restriction	No restriction	Restriction
Pre-op	80 (30–100)	80 (30–100)	2 (0–5)	2 (0–5)	39%	33%	35%	52%	55%	72%
Immediate post-op					36%	33%	8%	6%	54%	61%
3 months post-op	90 (40–100)	80 (50–100)	1 (0–4)	2 (0–4)	27%	23%	8%	8%	39%	61%
6-months post-op	80 (30–100)	80 (30–100)	2 (0–5)	2 (0–5)	23%	23%	11%	8%	34%	50%
Sig	0.289 (time 0.526)		0.541 (time 0.609)		0.654 (time 0.1)		0.927 (time 0.001)		0.226 (time = 0.220)	

Multivariate analysis generalized linear models, adjusted for temporal lesions.

*KPS* Karnofsky Performance Status, *MRS *Modified Rankin Scale.

*Speech deficits analysis was performed in a subgroup of dominant side surgeries (n = 103), comparing restriction (n = 18) vs non-restriction groups (n = 85).

#### Speech deficits

Speech deficits were analyzed in a subgroup of patients that underwent surgeries for tumors located in the dominant hemispheres (33 LGG and 103 GBM patients). We found a significantly higher rate of LGG patients with speech deficits in the post-op follow-up period in the restriction group, as compared to the non-restriction group (p = 0.004). All rim-restriction cases with speech deficits (n = 7) involved eloquent areas: either the left frontal, temporal or insular lobes. The percentage of patients with speech deficits in the post-op period decreased from 63 to 46% in the restriction group (p = 0.873) and from 18 to 9% (p = 0.989) in the non-restriction group after 1 year of follow-up, Table 3. Among GBM patients, no significant differences were noted in the rate of speech deficits in the post-op follow-up period between the restriction and non-restriction groups (p = 0.226), Table 4.

#### Functional assessment scores (KPS, MRS and cognitive tests)

We did not detect any significant differences in the KPS and MRS scores in either the LGG or GBM populations during our follow-up period (p > 0.05, see Tables 3 and 4).

Cognitive function tests, as previously described^11^, were performed in 33 LGG patients before and 3 months after surgery. We compared the results of 6 patients with post-op rim restriction and 27 with no restriction and could not detect any significant differences between the 2 groups in any of the functions assessed. Pre- and post-operative global cognitive scores were 98 ± 11and 95 ± 9 in the restriction group, and 95 ± 9 and 95 ± 9 in the non-restriction group, respectively (p = 0.194).

## Discussion

To the best of our knowledge, this is the first study to specifically describe the incidence as well as the short- and long-term clinical correlations of a phenomenon, defined as rim restriction on postoperative imaging following resection of low-grade gliomas and GBM, while excluding cases that got complicated by ischemic stroke. The relevance of this data lies in the question whether this quite prevalent abnormal post-operative imaging finding is linked to certain intra-operative parameters and can predict surgical outcomes.

Rim restriction was seen on the postoperative DWI studies in 32% and 23% of LGG and GBM patients, respectively. The incidence rates of postoperative rim restriction reportedly ranged from 16 to 39%, yet previous studies encompassed various types of tumors and also included patients that sustained intra-operative ischemic strokes, which as well present with restrictive changes on post-operative DWI.^12,13,23^

We found that the duration of surgery was significantly longer among patients that developed rim restriction on postoperative imaging as opposed to those without (Table 2) and these differences were particularly noted in the LGG group. Proposed mechanisms for the development of postoperative DWI restrictive changes included local intraoperative brain ischemia by direct vascular damage, coagulation, or vasospasm, as well as kinking of small arteries or mechanical tissue pressure by brain retraction^24,25^. Prolonged brain retraction is a potential cause for rim restriction in surgeries of longer duration and minimizing retraction might reduce its occurrence^9,24–26^.

We found that rim restriction is more common after surgeries for GBM involving the temporal lobe (p = 0.025) and tends to be higher in LGG surgeries involving the insula (p = 0.09). Previous reports demonstrated a higher risk for restrictive changes on DWI as well as for ischemic complications in insular region surgeries^13^. The insula is mainly supplied by frequent perforating arteries with no collateral flow, and surgeries in this location often require prolonged brain retraction^13,24–28^. As we often use retractors during trans-cortical resections of either temporal or insular gliomas, we raise the possibility that even mild surgical retraction in a diseased temporal lobe might be enough to induce the micro-ischemic changes which are often seen post-op. We did not detect any additional peri-operative parameters to be associated with the occurrence of rim restriction, including previously reported ones, such as recurrent operations and decreased MAP or IOM abnormalities (Tables 1 and 2).^15,25,26,29^Findings of rim restriction have been associated with evidence of remnant blood products in the surgical cavity on postoperative imaging, yet no significant association were seen in our study^9,30^.

We found a higher rate of speech deficits among LGG patients with rim restriction after they had undergone dominant-side surgeries in either the frontal, temporal or insular lobes, even after adjusting for the duration of surgery. (Table 3). It was previously reported that rim-pattern DWI abnormalities were non-significantly more common among patients with new postoperative neurological deficits (35%), as opposed to those without (25%), p = 0.177^10^. However, most studies viewed this imaging pattern as displaying either normal postoperative changes or as part of a larger group of ischemic complications, which did not enable subgroup analyses^9,13,15,23^. It is possible that rim restriction in dominant-side surgeries, particularly insular cases, is an indicator for subtle parenchymal injuries during surgery that manifest in speech deficits. Such injuries may be related to retraction forces applied on the tissue, as discussed above^24,27,28^, which seem to lead to a long-lasting damage, as there was no significant decrease in the rate of speech deficits over time. The GBM population showed a significant decrease in the rate of seizures following surgical resection, and these results are in line with previous reports regarding the beneficial effects of surgery in alleviating seizures among glioma patients, yet no differences were noted in relation to post-op restriction status.^31,32^.

Interestingly, the GBM population showed a unique yet non-significant trend towards increased overall and progression-free survival among patients with post-op rim restriction (p = 0.076 and p = 0.065, respectively). We could not clearly explain this trend and additional research is needed in order to validate and explore these findings.

Glioma patients often sustain various preoperative cognitive abnormalities that may further deteriorate after surgery, depending upon the eloquence of tumor location. These functions often improve when analyzed up to 1 year after surgery^33,34^. With mild cognitive abnormalities having been related to small strokes in proximity to the resection cavity^35^, we now searched for any association between rim restriction and cognitive changes following surgery, yet our cohort was too small to detect significant differences between the groups.

Interestingly, a recent study on perilesional rim restriction after surgical removal of convexity meningiomas found that restriction thicker than 1 cm was associated with post-op neurological deficits that lasted more than 3 months, including motor and speech deficit and seizures, with the majority improving over time. Main risk factors for post-op restriction were increasing age, intra-operative blood loss, tumor location over the motor strip and pre-op peri-tumor edema^36^.

The main limitation of this study is its historical cohort design, which forced us to estimate and define the degree of several study parameters, such as MRS and KPS, based on the study patients’ medical records. In addition, in the case of LGG, the study follow-up period was too short (5 years) to reveal differences in long-term survival between groups of patients. Furthermore, molecular IDH status was not available for the entire study population, as it included patients treated between the years 2013–2017. Finally, this study was not large enough to perform certain subgroup analyses, such as cognitive changes following dominant-side surgeries.

## Conclusions

Rim restriction on postoperative imaging is associated with longer durations of glioma surgeries, particularly of LGG, and more often occurs in cases of temporal and insular tumors. These imaging findings generally showed no apparent direct clinical consequences in either LGG or GBM, but they might be linked to a higher rate of speech deficits in dominant-side LGG surgeries.

## Study population and methods

### Study population

Our historical cohort included patients who underwent resection of LGG and GBM between January 2013 to December 2017. Preoperative clinical and intraoperative monitoring data were retrieved and documented, as were short- and long-term clinical outcomes. We selected patients that underwent surgical resection of LGG (World Health Organization 1 or 2) or GBM (WHO 4), and who had a full radiological and clinical dataset. The 2016 WHO classification became available in the middle of our study follow-up period (2013–2017). Due to the high ambiguity of WHO grade 3 that occasionally overlaps with grade II, but often tends to be more aggressive and overlap with grade IV, we decided not to include patients with the diagnosis of anaplastic astrocytoma in order to keep both study groups as uniform as possible. Patients who only underwent biopsies and those without full radiological and clinical data were excluded. In addition, in order to specifically focus on peri-resection rim restriction on post-operative imaging, we excluded patients who sustained intraoperative ischemic stroke (30 GBM and 19 LGG patients). The most recent surgery was considered as the index one in cases of patients who underwent more than one operation during the study period. This study was approved by the Tel-Aviv Sourasky Medical Center institutional ethics committee, reference number: 0768-17-TLV.

### Clinical and demographic data

We reviewed admission, surgical, and discharge reports for each case. Clinical data on follow-up in the ambulatory or hospital setting up to one year after surgery were also collected. The following clinical and demographic pre-and post-operative parameters were analyzed: age, sex, body mass index, hand dominance, other malignancies, brain radiotherapy, cerebrovascular, cardiac and metabolic comorbidities, and recurrent tumors, Karnofsky Performance Status (KPS) and Modified Rankin Score (MRS). Mean overall and progression free survival were also measured. Neurological manifestations were documented, including motor and speech deficits as well as report of seizures before and immediately (within hours) after surgery, and at 3, 6, and 12 months of follow-up, when available. Due to the inherent prognostic differences between each pathology groups, these outcomes were measured separately, up to 6 months in GBM patients and up to 12 months among LGG patients.

### Radiological data

**Figure 1:**
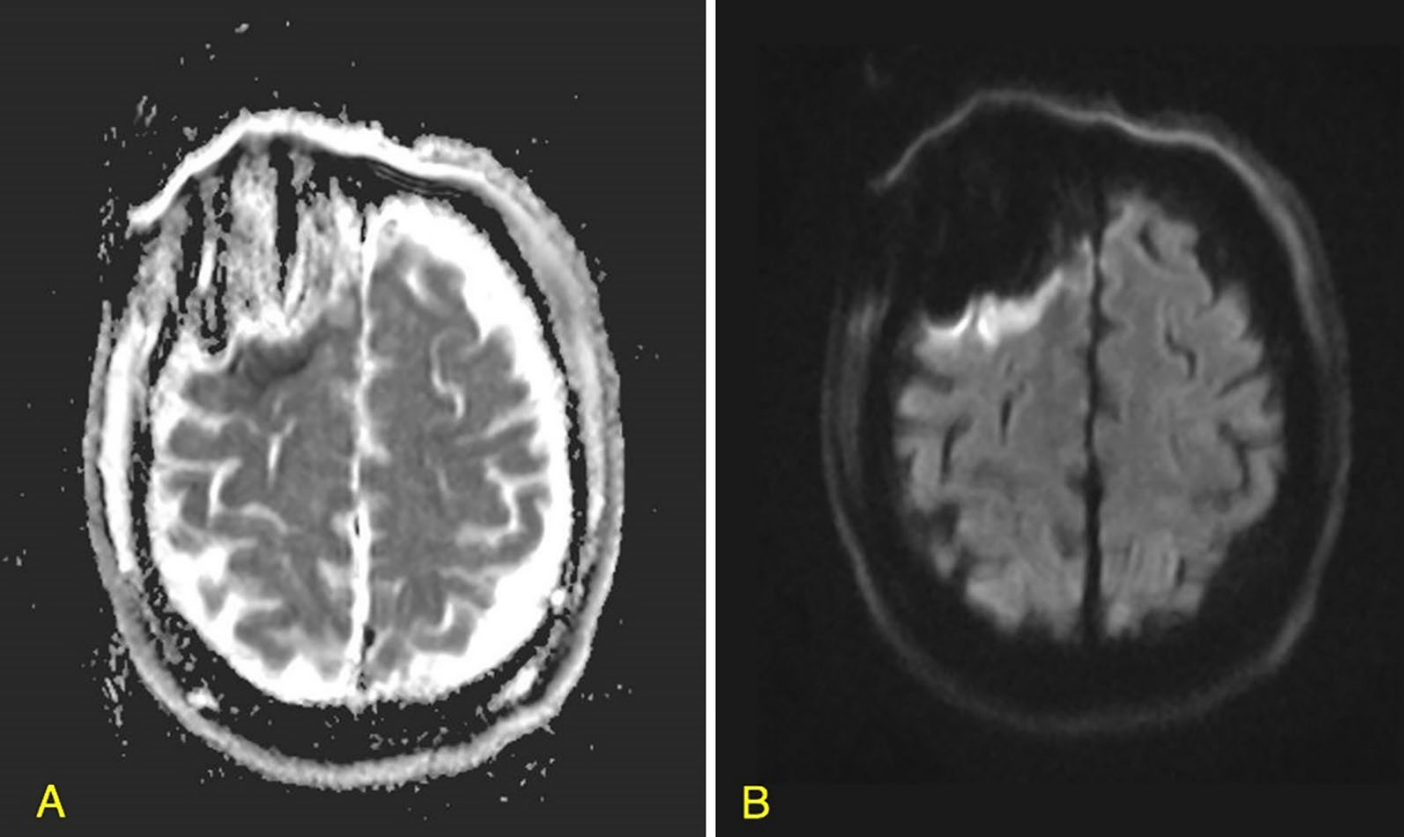
Post-up MRI imaging of a 52 year old patient after resection of GBM, with peri-resection rim restriction and no post-op neurological deficits. (**A**) Apparent-diffusion coefficient (ADC) sequence; (**B**) diffusion-weighted imaging (DWI) sequence.

Data on tumor volume and location, tumor enhancement, and extent of resection (EOR) were derived from pre- and immediate postoperative MRI studies, which are routinely performed within 48 h after surgery.^3,16^ . The EOR for GBM and LGG was calculated using the following formula: (preoperative − postoperative tumor volume)/preoperative tumor volume × 100). In case of GBM, the volume of blood products rather than the volume of the residual tumor was confirmed by comparing T1-weighted gadolinium-enhanced and non-enhanced MRIs. FLAIR sequence was used for measuring the non-enhancing component of the tumors^17^. Rim-pattern restriction surrounding the resection cavity was detected by a neuroradiologist who was blinded to clinical outcomes, and it was based on DWI studies and apparent diffusion coefficient techniques (Fig. 1). Infarcts, which were excluded from this study, were distinguished by their typical wedge-shaped arterial territory and relatively rapid appearance on immediate post-op DWI. Areas of T1-weighted hyper-intensities, accompanied by areas of hyperdensity on post-operative computerized tomographs (CT) were considered as blood products^11,14^.

### Neurocognitive analysis

LGG patients were evaluated by a battery of computerized cognitive tests before surgery and at the 3-month follow-up (Table 5). The NeuroTrax testing platform was used for the assessment of various cognitive functions, including visual and verbal memory, executive function, attention, naming, and visual spatial processing as was previously described^11,18–20^. Test scores were calculated by the software normalized for age and education level relative to a large normative database of healthy individuals and fit to an IQ-style scale, with higher scores reflecting better performance (mean:100, SD: 15).

**Table 5:** NeuroTrax tests and cognitive domains assessed in this study.

Verbal memory Non-verbal memory	Memory	Ten pairs of words are presented in four repetitions, each followed by a recognition test in which one member of a previously presented pair appears together with a list of four candidates for the other member of the pair. An additional recognition test is administrated following a delay Eight geometric objects are presented in four repetitions, each followed by a recognition test. Participants are required to remember the orientations of the originally objects. An additional recognition test is administered following a delay	Accuracy learning rate Accuracy learning rate
Verbal function	Verbal function	Pictures of common objects are presented. Participants are instructed to select the word that best rhymes with the name of the object. In the matching phase, participants are instructed to select the name of the object from four choices	Accuracy
Visual spatial processing	Visual spatial	Participants are instructed to imagine viewing a scene from the vantage point of a red pillar and choose from four alternative perspectives	Accuracy
Problem solving	Abstract reasoning/non-verbal IQ	Pictorial puzzles of gradually increasing difficulty are presented. Participants must choose the element that best completes the matrix from six possible alternatives	Accuracy

### Intraoperative neurophysiologic and anesthetics data

Changes in transcranial and direct cortical and subcortical motor evoked potentials (MEPs) of intraoperative monitoring (IOM) were evaluated as reported by our group^3,16,21,22^. Monitoring during awake craniotomies was performed by a trained neuropsychologist and it was based on continuous physical examination for motor functions, as well as language assessments for the detection of production and comprehension decline. Data on anesthetics included anesthesia duration and blood pressure measurements before and during surgery.

### Statistical analysis

Characteristics of categorical data were compared using the Pearson’s χ^2^ test. Multivariate logistic regression analysis was used to evaluate risk factors for the occurrence of rim restriction. Overall and progression free survival (PFS) were analyzed based on the Kaplan–Meier method. Repetitively measured variables were analyzed using generalized linear models. Statistics were performed using SPSS 21.0 software (SPSS Inc, Chicago, IL).

### Ethics approval

This study was approved by the Tel-Aviv Sourasky Medical Center institutional ethics committee, reference number: 0768-17-TLV. The study was performed in accordance with the relevant guidelines and regulations.

### Consent to participate

The ethics committee of the Tel-Aviv Sourasky Medical Center waived the requirement of informed consent due to the retrospective nature of the study.

## Data Availability

The datasets generated during and/or analyzed during the current study are available from the corresponding author on reasonable request. In the future we may consider asking our patients for their permission to share clinical data for research puropses.
